# Dieulafoy Lesion in the Ascending Colon Presenting with Gastrointestinal Bleeding and Severe Anemia Complicated by a Coexisting Severe Resistant Chronic Idiopathic Thrombocytopenic Purpura

**DOI:** 10.1155/2014/203678

**Published:** 2014-10-23

**Authors:** Sherif Ali Eltawansy, Brag Thyagarajan, Nadeem Baig

**Affiliations:** ^1^Department of Internal Medicine, Monmouth Medical Center, 300 Second Avenue, Long Branch, NJ 07740, USA; ^2^Section of Gastroenterology, Monmouth Medical Center, Long Branch, NJ 07740, USA

## Abstract

*Background*. GI (gastrointestinal) bleeding can be due to a variety of etiologies ranging from being common like bleeding peptic ulcer disease or esophageal varices. One of the rarely documented causes is the Dieulafoy lesion which is known as an abnormally large ectatic artery that penetrates the gut wall, occasionally eroding through the mucosa causing massive bleeding. In addition to that, we refer to the uncommon presentation of Dieulafoy lesion itself as it is well known to be found in the stomach, esophagus, duodenum, and jejunum but not the ascending colon as in our case. The patient had a coexisting ITP (idiopathic thrombocytopenic purpura) that was resistant to different therapies. *Case Report*. We report a case of a 48-year-old Egyptian female known for chronic ITP resistant to treatment. The patient presented with bright red bleeding per rectum and severe life threatening anemia. Endoscopic study showed a Dieulafoy lesion. Endoscopic clipping was successful in controlling the bleeding. *Conclusion*. Dieulafoy lesion is a rare reason for GI bleeding and can present in common or unexpected places. Also extreme caution should be used in patients with bleeding tendency due to different reasons, like ITP in our case.

## 1. Learning Objective

GI bleeding can be due to rare reasons like a Dieulafoy lesion which also can be found in unexpected sites for that kind of lesions. Extreme caution should be considered in patients with bleeding tendency disorders. Endoclipping is considered a successful treatment option for a Dieulafoy lesion.

## 2. Case Presentation

This is a case of 43-year-old Egyptian female with a past medical history of ITP resistant to steroid therapy that ended with splenectomy and history of hepatitis C and diabetes mellitus who comes to the ED (emergency department) for bright red bleeding per rectum for four days prior to presentation.

Approximately five years ago, our patient was diagnosed with ITP. She was treated with steroids and IVIG (*intravenous immunoglobulin*) and has had multiple blood and platelet transfusions for resistant ITP and thrombocytopenia that was reaching the levels of 10 to 20 × 10^9^/L. She was manifesting at that time by epistaxis and bleeding gums. She eventually had a splenectomy four years ago for refractory ITP. Our patient was diagnosed with hepatitis C which was presumed to be secondary to multiple blood transfusions. Since her splenectomy, platelet count was in the normal range till it started to drop again and oral steroid was resumed; then oral azathioprine was added. She developed steroid-induced hyperglycemia/diabetes mellitus.

Approximately 4 months ago, our patient had bright red blood per rectum and had an EGD (*esophagogastroduodenoscopy*) and colonoscopy which were unremarkable. Her platelet count was 12 × 10^9^/L during that time. She was transfused with platelets and packed red blood cells in addition to resuming steroids. The capsule endoscopy was suggested by the treating physician but was not done as it was not available for the patient as she was staying in a rural area in Egypt and lost follow-up with the hospital for financial issues; then she travelled to USA.

Four days before the current admission, our patient had started having bright red bleeding per rectum which was 3-4 times a day. Bleeding was associated with colicky abdominal pain. No other orificial bleeding was appreciated. Her medications on last admission included tranexamic acid tablets, ethamsylate tablets, prednisone 20 mg tablets, and azathioprine 100 mg tablets. The patient has never smoked or had alcohol in the past and had no history of illicit drug use. She is a housewife by occupation.

In the ED on admission, the patient was vitally stable. Blood work shows hemoglobin of 350 g/L and platelets of 108 × 10^9^/L.

The patient was admitted to the intensive care unit for close monitoring. Omeprazole drip and octreotide drip were administered. There were no further episodes of bleeding and her hemoglobin improved to 680 g/L in the following 2 days.

**Figure 1:**
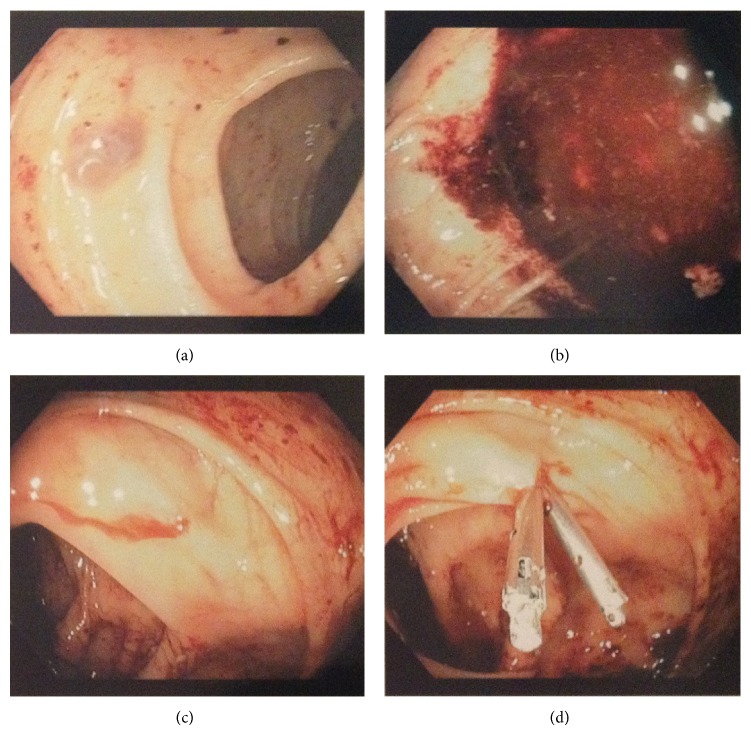
Procedure: Olympus colonoscope was advanced from the anus under direct visualization to the cecum. Cecum was confirmed by appendiceal orifice and ileocecal valve. There was large amount of clots and blood throughout the colon, and there was active bleeding from the proximal ascending colon. After irrigation it appeared to be from a Dieulafoy lesion at the proximal ascending colon with active bleeding. Two endoclips were placed with excellent hemostasis. It was decided not to use cautery due to the patient's thrombocytopenia. Due to the presence of large amount of old blood and clots throughout the colon, polyps cannot be ruled out, so this procedure was not optimal for screening purposes. The patient tolerated the procedure very well and there was no immediate complication associated with the procedure.

The endoscopy showed small hiatal hernia, mild gastritis, and no evidence of esophageal or gastric varices. The colonoscopy revealed an actively bleeding Dieulafoy lesion (Figure 1) in her ascending colon. Two endoclips were placed on the Dieulafoy lesion and excellent hemostasis was obtained. Clear liquid diet was started later, the omeprazole and octreotide drips were discontinued, and she was transfused with a total of 7 units of packed RBCs by the end of hospital stay beside 2 units of platelets transfusion. By the end of 7-day hospital stay which was between the ICU and the regular floor, hemoglobin went up to 930 g/L and platelet went up to 53 × 10^9^/L.

The patient did not have any new bleeding per rectum after the surgical clipping of the Dieulafoy lesion and never dropped to the first hemoglobin level (350 g/L) that was on admission.

On the seventh day of admission, the patient had a repeat colonoscopy which revealed both endoclips in position with no new bleeding. There was trace oozing of blood and hemostasis was attained with 1 cc of 1 : 10,000 epinephrine and cautery.

Hemoglobin was 114 g/L and platelet count was 144 × 10^9^/L two weeks later after the discharge and follow-up in the clinic. The patient did not develop any new GI bleeding.

## 3. Discussion

Our case represents an example of an acute GI bleeding secondary to a rare lesion called Dieulafoy's lesion. Dieulafoy's lesion by definition is a submucosal ectatic artery in the gastrointestinal tract. Dieulafoy's lesion was first described by Gallard [1] in 1884 and later named after the French surgeon Dieulafoy [2]. It is larger than the vessels usually in that area. It can occur in any part of the GI tract, although most frequently it is in the stomach. The etiology of these lesions is still unknown and usually the artery is nonulcerated [3]. The incidence of Dieulafoy lesion leading to GI hemorrhage ranges from 0.5% to 14%, depending upon the study. It is more common in men and presents at around 50 years of age [4]. Approximately 75% to 95% of Dieulafoy lesions are found within 6 cm of the gastroesophageal junction, predominantly on the lesser curve [5]. The blood supply to that portion of the stomach is from a large submucosal artery arising directly from the left gastric artery. It has been suggested that the thin mucosa overlying a pulsating artery is eroded progressively by the mechanical pressure from the abnormal vessel [5]. Lesions of similar morphological and histological features have been found in the distal esophagus [6], the duodenal bulb [7], the jejunum [8, 9], the colon [10–12], and the rectum [13, 14]. Different theories were postulated about the etiology of that lesion and if it can be congenital or acquired vascular malformation but it is thought that the artery protrudes through a solitary, tiny mucosal defect (2–5 mm), commonly in the upper part of the stomach [15, 16]. It may rupture spontaneously and lead to massive bleeding [17, 18].

Histologically, the eroded artery appears normal. There is no evidence of any mucosal inflammatory process, signs of deep ulcerations, penetration of the muscularis propria, vasculitis, aneurysm formation, or arteriosclerosis [19].

The most common presenting symptom is recurrent, often massive, hematemesis associated with melena (51%). The lesion may present with hematemesis alone (28%) or melena alone (18%) [20]. The mean hemoglobin level on admission had been reported to be between 840–920 g/L in various studies. The average transfusion requirement for the initial resuscitation is usually in excess of three [20] and up to 8 units of packed red blood cells [21–23]. It is important to mention that our patient had a hemoglobin level of 350 g/L on admission and received 7 units of packed RBCs units to raise the hemoglobin level till it became 930 g/L on discharge from the hospital after 8-day stay in the hospital.

Therapeutic endoscopy has been used successfully and is now the modality of choice for the initial treatment of Dieulafoy lesions [21–23]. Endoscopic modalities used include bipolar electrocoagulation, monopolar electrocoagulation, injection sclerotherapy, heater probe, laser photocoagulation, epinephrine injection, hemoclipping, and banding [21–23]. The injection of epinephrine has been used in combination with other modalities, as a means to slow or stop bleeding and allow better visualisation of the lesion and successful treatment [24]. Our case was successfully managed with endoclipping of the identified lesion. The bleeding lesion was well controlled with 2 endoclips and cautery was avoided due to severe thrombocytopenia. A second colonoscopy was performed 4 days later to follow the Dieulafoy lesion and there was minor bleeding from the lesion that was controlled with 1 mL of diluted epinephrine in 1 : 10,000 solution, injected into the base, and a 10-French BICAP cautery was applied to the site.

In summary, our case represents a 48-year-old female with a low GI bleeding that was complicated by severe anemia (350 g/L hemoglobin on admission). As we mentioned, Dieulafoy lesion is an uncommon finding and it is rare to be found in the ascending colon as it was seen on the colonoscopy close to the cecum. Dieulafoy lesion is known to be ominous for severe GI bleeding that can lead to circulatory collapse and severe blood loss requiring massive blood transfusion sometimes [25] and combining that with the fact that the patient has resistant ITP warrants severe caution in dealing with a case with these comorbidities. The patient was on prednisone and azathioprine and due to the persistent severe thrombocytopenia, the patient was admitted to hospital 4 years ago where high dose steroid and IVIG (*intravenous immunoglobulin*) were tried. Splenectomy was eventually done. Still the ITP was not well controlled on follow-up. Platelet count on this admission was found to be low down to 40 × 10^9^/L. The patient also has a history of chronic hepatitis C that was proven by liver biopsy done 4 years ago. There was no evidence of liver cirrhosis during this admission or the previous admission 4 years ago where the patient was evaluated for the splenectomy at that time to control the ITP. Upper GI endoscopy at that time excluded portal hypertension and esophageal varices.

It is worthy to mention also that diagnosis of the Dieulafoy lesion may need multiple endoscopies till the bleeding source is identified [26]. Our case had a previous colonoscopy with esophagogastroduodenoscopy 4 months prior to the presentation to us and did not reveal any similar lesions at that time. Camera capsule was planned but not done due to financial and social barriers.

Follow-up of the patient in the clinic 2 weeks after discharge was reassuring with no new clinical bleeding. Blood work showed hemoglobin of 114 g/L and platelet count was 144 × 10^9^/L. The patient was suffering from severe vitamin D deficiency due to the long standing steroid dose to control ITP.

## 4. Conclusion

GI bleeding can be challenging and found to be secondary to rare lesions like a Dieulafoy lesion that requests close follow-up due to the its nature of presenting with severe bleeding with possible recurrence specially in those with bleeding tendency disorders.
